# Prevalência sorológica de infecção por SARS-CoV-2 entre trabalhadores do sistema prisional do Espírito Santo, 2020

**DOI:** 10.1590/S1679-49742022000100008

**Published:** 2022-02-28

**Authors:** Camila Leal Cravo Duque, Laylla Ribeiro Macedo, Ethel Leonor Noia Maciel, Ricardo Tristão-Sá, Erika do Nascimento Bianchi, Adriana Ilha da Silva, Pablo Medeiros Jabor, Cristiana Costa Gomes, Orlei Amaral Cardoso, Pablo Lira, Raphael Lubiana Zanotti, Silvânio José de Souza Magno, Eliana Zandonade

**Affiliations:** 1 Secretaria de Estado da Justiça do Espírito Santo, Diretoria de Saúde do Sistema Penal, Vitória, ES, Brasil Secretaria de Estado da Justiça do Espírito Santo Diretoria de Saúde do Sistema Penal Vitória ES Brasil; 2 Universidade Federal do Espírito Santo, Laboratório de Epidemiologia, Vitória, ES, Brasil Universidade Federal do Espírito Santo Universidade Federal do Espírito Santo Laboratório de Epidemiologia Vitória ES Brazil; 3 Universidade Federal do Espírito Santo, Departamento de Medicina Social, Vitória, ES, Brasil Universidade Federal do Espírito Santo Universidade Federal do Espírito Santo Departamento de Medicina Social Vitória ES Brazil; 4 Instituto Jones dos Santos Neves, Coordenação de Geoespacialização, Vitória, ES, Brasil Instituto Jones dos Santos Neves Coordenação de Geoespacialização Vitória ES Brasil; 5 Secretaria de Estado da Saúde do Espírito Santo, Subsecretaria de Estado de Vigilância em Saúde, Vitória, ES, Brasil Secretaria de Estado da Saúde do Espírito Santo Subsecretaria de Estado de Vigilância em Saúde Vitória ES Brasil; 6 Instituto Jones dos Santos Neves, Diretoria de Integração e Projetos Especiais, Vitória, ES, Brasil Instituto Jones dos Santos Neves Diretoria de Integração e Projetos Especiais Vitória ES Brasil; 7 Secretaria de Estado da Justiça do Espírito Santo, Subsecretaria de Planejamento e Controle, Vitória, ES, Brasil Secretaria de Estado da Justiça do Espírito Santo Subsecretaria de Planejamento e Controle Vitória ES Brasil; 8 Universidade Federal do Espírito Santo, Departamento de Estatística, Vitória, ES, Brasil Universidade Federal do Espírito Santo Universidade Federal do Espírito Santo Departamento de Estatística Vitória ES Brazil

**Keywords:** Prevalência, Infecções por Coronavírus, Prisões, Saúde do Trabalhador, Estudos Transversais, Prevalence, Coronavirus Infections, Prisons, Occupational Health, Cross-Sectional Studies., Prevalencia, Infecciones por Coronavirus, Prisiones, Salud Laboral, Estudios Transversales

## Abstract

**Objetivo:**

Estimar a prevalência de infecção por SARS-CoV-2 entre trabalhadores do sistema prisional do Espírito Santo, Brasil, no período agosto-setembro de 2020.

**Métodos:**

Inquérito em amostra estratificada, mediante entrevistas e testes sorológicos para SARS-CoV-2.

**Resultados:**

Nos 986 pesquisados, a prevalência sorológica de infecção por SARS-CoV-2 foi de 11,9% (IC_95%_ 8,1%;15,7%) nos profissionais de saúde, e de 22,1% (IC_95%_ 18,8%;25,3%) nos agentes penitenciários. A positividade foi mais frequente nos profissionais da saúde do norte do estado (19,7%) e em agentes penitenciários do sexo masculino (24,0%). Entre soropositivos, a fadiga foi o sintoma mais frequente nos agentes penitenciários (13,4%), e a mialgia, nos profissionais de saúde (10,8%); e as comorbidades mais prevalentes entre os positivos foram asma ou bronquite (16,2%), para profissionais de saúde, e hipertensão para agentes penitenciários (12,8%).

**Conclusão:**

A prevalência sorológica de SARS-Cov-2 foi maior nos agentes penitenciários, achado que deve subsidiar ações de controle e prevenção da doença nesse cenário.

## Abstract (en)

**Objective:**

To estimate the serological prevalence of SARS-CoV-2 infection among prison system workers in the state of Espírito Santo, Brazil, between August-September 2020.

**Methods:**

This was a stratified sample survey, using interviews and serological tests for SARS-CoV-2.

**Results:**

Among the 986 interviewers, the serological prevalence of SARS-CoV-2 infection was 11.9% (95%CI 8.1%;15.7%) in health professionals, and 22.1% (95%CI 18.8%;25.3%) in prison officers. Positivity was more frequent among health professionals in the north of the state (19.7%) and in male prison officers (24.0%). Among seropositive individuals, fatigue was the most frequent symptom in prison agents (13.4%) and myalgia in health professionals (10.8%); and the most prevalent comorbidities among the seropositive individuals were asthma or bronchitis (16.2%), in health professionals, and hypertension in prison officers (12.8%).

**Conclusion:**

The serological prevalence of SARS-Cov-2 infection was higher in prison officers, a finding that can support disease control and prevention actions in this scenario.

## Resumen

**Objetivo:**

Estimar la prevalencia serológica de la infección por SARS-CoV-2 en trabajadores del sistema penitenciario de Espírito Santo, Brasil, entre agosto y setiembre de 2020.

**Métodos:**

Encuesta muestral estratificada mediante entrevistas y pruebas serológicas para SARS-CoV-2.

**Resultados:**

En los 986 encuestados, la prevalencia serológica de la infección por SARS-CoV-2 fue del 11,9% (IC_95%_ 8,1%;15,7%) entre los profesionales de la salud y del 22,1% (IC_95%_ 18,8%;25,3%) entre los funcionarios de prisiones. La positividad fue más frecuente en los profesionales de la salud de la Región Norte (19,7%) y en los funcionarios de prisiones varones (24,0%). Entre los positivos, la fatiga fue el síntoma más frecuente entre los funcionarios de prisiones (13,4%) y la mialgia entre los profesionales de la salud (10,8%); y las comorbilidades más prevalentes entre las positivas fueron: asma o bronquitis (16,2%) para los profesionales de la salud e hipertensión para los funcionarios de prisiones (12,8%).

**Conclusión:**

La prevalencia serológica del SARS-Cov-2 fue mayor en los funcionarios de prisiones, apoyando las acciones de control y prevención de enfermedades en este escenario.

## INTRODUÇÃO

**Table:** 

Contribuições do estudo	
Principais resultados	Nos 986 trabalhadores do sistema prisional do Espírito Santo pesquisados, a prevalência sorológica de infecção por SARS-CoV-2 foi 11,9% (IC_95%_ 8,1%;15,7%) nos profissionais de saúde, e 22,1% (IC_95%_ 18,8%;25,3%) nos agentes penitenciários.
Implicações para os serviços	O conhecimento dos indicadores sorológicos relacionados aos trabalhadores do ambiente prisional oportuniza aos gestores dessas instituições o direcionamento e a qualificação de ações com vistas ao enfretamento da pandemia da COVID-19.
Perspectivas	Reflexões sobre as fragilidades do sistema penal podem contribuir para a melhoria das condições sanitárias observadas nos espaços prisionais, evidenciando a necessidade de zelar pela manutenção da saúde e do bem-estar dos trabalhadores desses ambientes.

Após um ano da declaração da Organização Mundial da Saúde (OMS) de reconhecimento da pandemia de COVID-19, em março de 2020, observa-se que alguns grupos populacionais, a exemplo de negros e indivíduos com menores renda e escolaridade, têm-se mostrado mais vulneráveis aos óbitos pelo SARS-CoV-2.^1^^,^^2^

Além disso, indivíduos em ambientes confinados e com potencial aglomeração de pessoas estão mais expostos ao contágio e à disseminação do vírus.^3^ Nesse sentido, o ambiente prisional oferece condições que, potencialmente, facilitam a propagação da COVID-19,^3^ haja vista a precária situação em que se encontram as instituições penais brasileiras, descritas como ambientes malconservados, com pouca ou nenhuma ventilação, além do problema crônico de superlotação.^4^

Os dados do Conselho Nacional de Justiça (CNJ), divulgados pelo Departamento Penitenciário Nacional (Depen), mostram que não somente a população privada de liberdade (PPL) tem sido apontada como um grupo mais vulnerável ao acometimento da COVID-19, senão também os trabalhadores lotados nesses espaços, como profissionais de saúde; e de segurança, como agentes penitenciários e administrativos.^5^

Até o dia 22 de fevereiro de 2021, foram realizados 62.459 testes e diagnosticados 15.450 casos de COVID-19 em servidores do sistema prisional brasileiro, dos quais 112 chegaram a óbito. Entre as Unidades da Federação, São Paulo foi o estado que contabilizou maior número de casos (2.751), enquanto o Rio Grande do Sul teve a menor ocorrência.^13^ A região Sul do Brasil apresentou o menor percentual de óbitos relacionados à doença, 5,4%, e a região Sudeste respondeu por 46,6% das mortes por COVID-19 no país. No estado do Espírito Santo, região Sudeste, 835 casos em trabalhadores do sistema prisional e três óbitos pela doença haviam sido notificados até aquela data.^5^

Um estudo internacional sobre dados do Projeto COVID nas Prisões, referentes ao período entre abril de 2020 e janeiro de 2021, mostrou que a taxa de casos de COVID-19 nos funcionários dessas instituições foi superior àquela encontrada para a população geral, e próxima à observada na PPL dos países estudados.^6^

Dessa forma, torna-se de extrema importância conhecer essa realidade, para que as instituições de saúde e/ou segurança planejem estratégias visando controlar a transmissão da COVID-19 nesses ambientes e, consequentemente, proteger a saúde de pessoas privadas de liberdade, servidores penitenciários, profissionais de saúde, advogados ou profissionais da Justiça, ou qualquer indivíduo que ingresse nas unidades prisionais, além da comunidade geral.^7^

Este estudo teve por objetivo estimar a prevalência sorológica de infecção por SARS-CoV-2 entre trabalhadores do sistema prisional do estado do Espírito Santo, Brasil.

## MÉTODOS

Trata-se de um estudo transversal, realizado a partir de um inquérito de base populacional,^8^ com amostragem por cotas, em unidades prisionais do Espítiro Santo, estratificada de acordo com as categorias de trabalhadores ‘profissionais de saúde’ e ‘agentes penitenciários’.

O estudo foi realizado entre os dias 31 de agosto e 4 de setembro de 2020, e teve como população-alvo trabalhadores das 34 unidades prisionais do Espírito Santo, distribuídas em 13 munícipios do estado. Nessas unidades, atuavam 441 profissionais de saúde e 3.101 agentes penitenciários.

Para o cálculo da amostra, considerou-se o tamanho das populações descritas, uma prevalência esperada de COVID-19 de 10%, erro amostral de 2 pontos percentuais e nível de confiança de 5%. Os tamanhos mínimos definidos para a amostra foram de 293 profissionais da saúde e 667 agentes penitenciários, calculados proporcionalmente ao número de profissionais da saúde e agentes penitenciários de cada unidade prisional.

Uma semana antes do início da pesquisa, a partir de uma lista fornecida pela Administração do Sistema Prisional do Espírito Santo, realizou-se um sorteio aleatório dos nomes que fariam parte da pesquisa, considerando-se as duas categorias de trabalhadores. Nessa etapa do processo, incluiu-se em torno de 40% de participantes a mais, visando compensar eventuais faltas ou licenças, ou alternância de turnos de trabalho.

Foi desenvolvido um aplicativo no *software* Plataforma ArcGIS Online, para ser instalado em aparelhos celulares e utilizado de forma completamente *offline*, o que possibilitou a coleta de dados no interior das unidades prisionais. No final de cada dia de aplicação, os aparelhos eram conectados à internet e a equipe técnica recebia, imediatamente, o pacote de dados coletados naquela jornada.

Foi desenvolvido um painel de acompanhamento da pesquisa para cada uma das unidades prisionais, de modo a se monitorar quais unidades estavam próximas ou já haviam atingido as metas para cada um dos perfis abordados no inquérito - servidores penitenciários e profissionais da saúde do sistema prisional do estado.

Os entrevistadores foram treinados na execução do exame, que foi realizado com amostra de sangue colhida por punção digital. Utilizou-se o teste rápido imunocromatográfico de anticorpo, IgM e IgG, da marca MedLevensohn, com registro na Agência Nacional de Vigilância Sanitária (Anvisa) n^o^ 80560310056. O resultado foi computado a partir da observação dos marcadores, após o devido tempo indicado pelo fabricante. No caso de dúvidas de leitura, os testes foram repetidos e encaminhados para a equipe médica responsável pela pesquisa. Considerou-se o teste positivo - IgG ou IgM-; contudo, a informação relativa a qual dos dois marcadores teve resultado positivo não estava disponível para os autores.

A equipe de entrevistadores recebeu todos os equipamentos de proteção individual (EPIs) recomendados pela Agência Nacional de Vigilância Sanitária (Anvisa) para uma situação de possível contato com pessoas portadoras de COVID-19, além de outros materiais necessários, como telefones celulares e álcool, e demais equipamentos utilizados no inquérito sorológico, fornecidos pela Secretaria de Estado da Saúde do Espírito Santo.

Além da testagem para COVID-19, foram colhidas informações dos participantes mediante entrevista com perguntas fechadas, realizada face a face. Para tanto, foram selecionadas e categorizadas as seguintes variáveis:

- sexo (masculino; feminino);
- faixa etária (em anos: até 30; 31 a 40; 41 a 50; 51 e mais);
- escolaridade (ensino médio completo; ensino superior incompleto; ensino superior completo; mestrado);
- raça/cor da pele (amarela; branca; indígena; parda; preta);
- número de sintomas de COVID-19 nos últimos 15 dias (nenhum; 1; 2; 3 ou mais);
- acesso ao serviço de saúde nos últimos 15 dias (não; sim);
- hábito de higienização das mãos (não; sim);
- utilização de transporte público para deslocamento ao trabalho (não; sim);
- frequência de utilização diária de transporte público para deslocamento ao trabalho (não utiliza; até 3 vezes; 4 vezes ou mais);
- tempo médio gasto com o deslocamento diário em transporte público (não utiliza; menos de 30 minutos; entre 30 e 60 minutos; mais de 60 minutos); e
- carga horária de trabalho (em horas: 20; 30; 40).

As informações foram armazenadas em um banco de dados, e a análise estatística realizada utilizando-se o programa Statistical Package for the Social Science (SPSS), versão 20.0. Foram estimadas as prevalências, e respectivos intervalos de confiança de 95% (IC_95%_). As diferenças entre as variáveis de exposição e a soropositividade por COVID-19, estratificadas para profissionais de saúde e agentes penitenciários, foram analisadas utilizando-se o teste qui-quadrado de Pearson, ou o teste exato de Fisher quando apropriado. O nível de significância adotado foi de 5%.

Todos os indivíduos selecionados para a amostra do inquérito nas unidades prisionais foram informados sobre os objetivos do estudo, riscos e benefícios implicados, acompanhados de aconselhamento sobre medidas preventivas. O material para sorologia e as informações foram coletados após a assinatura do Termo de Consentimento Livre e Esclarecido pelos participantes. Todos os indivíduos testados receberam a informação do resultado do teste alguns minutos após sua realização. Os casos positivos foram notificados e encaminhados ao Serviço Municipal de Saúde, para avaliação clínica. Todas as medidas de biossegurança foram tomadas, de maneira a garantir a saúde dos trabalhadores de campo. O estudo foi aprovado pelo Comitê de Ética em Pesquisa com Seres Humanos, do Centro de Ciências da Saúde da Universidade Federal do Espírito Santo (CEP/CCS/UFES): Parecer n^o^ 4.209.127, emitido em 12 de agosto de 2020.

## RESULTADOS

Foram pesquisados 986 trabalhadores do sistema prisional do Espírito Santo: 311 profissionais de saúde e 675 agentes penitenciários. Não houve recusas desses profissionais à participação no estudo.

Os profissionais de saúde participantes atuavam, em sua maioria, em presídios localizados na região metropolitana de Vitória (61,7%), com regime de prisão fechado (90,7%), eram do sexo feminino (76,8%), da faixa etária entre 31 e 40 anos (41,3%), possuíam ensino superior completo (43,5%) e se declararam de raça/cor da pele parda (44,8%). Entre os agentes penitenciários, a maior parte atuava em unidades prisionais da mesma região metropolitana de Vitória (59,4%), com regime de prisão fechado (89,5%), eram do sexo masculino (76,1%), da faixa etária entre 31 e 40 anos (54,8%), possuíam ensino superior completo (45,5%) e se declararam de raça/cor da pele parda (49,9%) (Tabela 1).

**Tabela 1:** - Distribuição das características sociodemográficas de profissionais de saúde e agentes penitenciários, segundo o resultado do teste rápido para SARS-CoV-2, Espírito Santo, 2020

Variável	Categoria	Profissional de saúde N=311^a^				Agente penitenciário N=675^a^			
		Positivo	Negativo	Total	p-valor^b^	Positivo	Negativo	Total	p-valor^b^
		n (%)	n (%)	n (%)		n (%)	n (%)	n (%)	
Localização do presídio (região)									
	Metropolitana	14 (7,3)	178 (92,7)	192 (61,7)	0,006	86 (21,4)	315 (78,6)	401 (59,4)	0,178
	Norte	18 (19,7)	73 (80,3)	91 (29,3)		50 (25,9)	143 (74,1)	193 (28,6)	
	Sul	5 (17,9)	23 (82,1)	28 (9,0)		13 (16,0)	68 (84,0)	81 (12,0)	
Regime de prisão									
	Fechado	35 (12,4)	247 (87,6)	282 (90,7)	0,382^c^	139 (23,0)	465 (77,0)	604 (89,5)	0,086
	Aberto	2 (6,9)	27 (93,1)	29 (9,3)		10 (14,1)	61 (85,9)	71 (10,5)	
Sexo									
	Feminino	31 (13,0)	208 (87,0)	239 (76,8)	0,287	24 (15,0)	136 (85,0)	160 (23,9)	0,022
	Masculino	6 (8,3)	66 (91,7)	72 (23,2)		123 (24,0)	388 (76,0)	511 (76,1)	
Faixa etária (em anos)									
	≤30	13 (11,3)	102 (88,7)	115 (37,1)	0,543^c^	9 (19,1)	38 (80,9)	47 (7,0)	0,665
	31-40	14 (10,9)	114 (89,1)	128 (41,3)		77 (20,9)	292 (79,1)	369 (54,8)	
	41-50	9 (17,7)	42 (82,3)	51 (16,4)		50 (24,0)	158 (76,0)	208 (30,9)	
	≥51	1 (6,3)	15 (93,7)	16 (5,2)		13 (26,5)	36 (73,5)	49 (7,3)	
Escolaridade									
	Ensino médio completo	11 (20,0)	44 (80,0)	55 (17,8)	0,012^c^	52 (28,7)	129 (71,3)	181 (26,9)	0,025
	Ensino superior incompleto	3 (15,8)	16 (84,2)	19 (6,1)		15 (16,3)	77 (83,7)	92 (13,6)	
	Ensino superior completo	14 (10,4)	121 (89,6)	135 (43,5)		64 (20,9)	243 (79,1)	307 (45,5)	
	Mestrado	8 (7,9)	93 (92,1)	101 (32,6)		17 (18,1)	77 (81,9)	94 (14,0)	
Raça/cor da pele									
	Amarela	1 (33,3)	2 (66,7)	3 (1,0)	0,022^c^	(0,0)	4 (100,0)	4 (0,6)	0,472^c^
	Branca	7 (5,5)	120 (94,5)	127 (41,0)		45 (20,1)	179 (79,9)	224 (33,2)	
	Indígena	(0,0)	(0,0)	(0,0)		(0,0)	4 (100,0)	4 (0,6)	
	Parda	25 (18,0)	114 (82,0)	139 (44,8)		81 (24,0)	256 (76,0)	337 (49,9)	
	Preta	4 (9,7)	37 (90,3)	41 (13,2)		23 (21,7)	83 (78,3)	106 (15,7)	

a) Total de entrevistados; esse total pode diferir para algumas variáveis, devido a não resposta dos participantes; b) teste qui-quadrado de Pearson; c) teste exato de Fisher.

A prevalência de casos positivos foi de 11,9% (IC95% 8,1%;15,7%) entre os profissionais de saúde, e de 22,1% (IC95% 18,8%;25,3%) entre os agentes penitenciários.

Os casos positivos para SARS-CoV-2 foram mais frequentes nos profissionais de saúde da região norte do Espírito Santo (19,7%) e nos agentes penitenciários do sexo masculino (24,0%). Em ambos os grupos de profissionais, o desfecho mostrou-se associado a menor escolaridade (ensino médio completo) (Tabela 1).

A maioria dos profissionais de saúde e dos agentes penitenciários não apresentou sintomas (67,5% dos profissionais de saúde e 64,9% dos agentes penitenciários) e não havia procurado atendimento em serviços de saúde nos últimos 15 dias (77,5% dos profissionais de saúde e 83,8% dos agentes penitenciários), não apresentavam comorbidades (73,6% dos profissionais de saúde e 77,8% dos agentes penitenciários), relataram hábito de higienizar as mãos (99,0% dos profissionais de saúde e 98,2% dos agentes penitenciários), não utilizavam transporte público para deslocamento ao trabalho (61,0% dos profissionais de saúde e 90,1% dos agentes penitenciários) e possuíam carga horária de trabalho semanal de 40 horas (48,7% dos profissionais de saúde e 94,3% dos agentes penitenciários) (Tabela 2).

**Tabela 2:** - Distribuição do número de sintomas, comorbidades, procura por serviço de saúde e hábito de lavar as mãos, entre profissionais de saúde e agentes penitenciários, segundo o resultado do teste rápido para SARS-CoV-2, Espírito Santo, 2020

Variável	Categoria	Profissional de saúde N=311^a^				Agente penitenciário N=675^a^			
		Positivo	Negativo	Total	p-valor^b^	Positivo	Negativo	Total	p-valor^b^
		n (%)	n (%)	n (%)		n (%)	n (%)	n (%)	
Número de sintomas nos últimos 15 dias									
	Nenhum	24 (11,4)	186 (88,6)	210 (67,5)	0,342^c^	89 (20,3)	349 (79,7)	438 (64,9)	0,026
	1	7 (17,9)	32 (82,1)	39 (12,5)		20 (17,4)	95 (82,6)	115 (17,0)	
	2	1 (3,6)	27 (96,4)	28 (9,0)		15 (32,6)	31 (67,4)	46 (6,8)	
	3	1 (7,7)	12 (92,3)	13 (4,2)		10 (28,6)	25 (71,4)	35 (5,2)	
	≥4	4 (19,1)	17 (80,9)	21 (6,8)		15 (36,6)	26 (63,4)	41 (6,1)	
Acesso ao serviço de saúde nos últimos 15 dias									
	Não	30 (12,6)	208 (87,4)	238 (77,5)	0,580	126 (22,3)	439 (77,7)	565 (83,8)	0,782
	Sim	7 (10,1)	62 (89,9)	69 (22,5)		23 (21,1)	86 (78,9)	109 (16,2)	
Número de comorbidades									
	Nenhuma	23 (10,1)	206 (89,9)	229 (73,6)	0,150^c^	117 (22,3)	408 (77,7)	525 (77,8)	0,972^c^
	1	12 (18,8)	52 (81,2)	64 (20,6)		25 (21,4)	92 (78,6)	117 (17,3)	
	2	1 (6,7)	14 (93,3)	15 (4,8)		5 (21,7)	18 (78,3)	23 (3,4)	
	3	1 (33,3)	2 (67,7)	3 (1,0)		1 (14,3)	6 (85,7)	7 (1,0)	
	≥4	- (0,0)	- (0,0)	- (0,0)		1 (33,3)	2 (66,7)	3 (0,5)	
Hábito de higienização das mãos									
	Não	- (0,0)	3 (100,0)	3 (1,0)	0,521^c^	1 (8,3)	11 (91,7)	12 (1,8)	0,247^c^
	Sim	37 (12,1)	269 (87,9)	306 (99,0)		148 (22,3)	515 (77,7)	663 (98,2)	
Utilização de transporte público para o deslocamento ao trabalho									
	Não	24 (12,7)	165 (87,3)	189 (61,0)	0,817	130 (21,4)	477 (78,6)	607 (90,1)	0,101
	Sim	13 (10,7)	108 (89,3)	121 (39,0)		18 (26,9)	49 (73,1)	67 (9,9)	
Frequência de utilização diária de transporte público para deslocamento ao trabalho									
	Não utiliza	24 (12,6)	166 (87,4)	190 (61,1)	0,812^c^	131 (21,6)	477 (78,4)	608 (90,1)	0,178
	4 vezes ou mais	10 (11,5)	77 (88,5)	87 (28,0)		9 (20,9)	34 (79,1)	43 (6,4)	
	Até 3 vezes	3 (8,8)	31 (91,2)	34 (10,9)		9 (37,5)	15 (62,5)	24 (3,5)	
Tempo médio com o deslocamento diário em transporte público									
	Não utiliza	24 (12,6)	166 (87,4)	190 (61,1)	0,641^c^	131 (21,6)	477 (78,4)	608 (90,1)	0,506^c^
	Menos de 30 min.	1 (6,7)	14 (93,3)	15 (4,8)		3 (37,5)	5 (62,5)	8 (1,2)	
	Entre 30 e 60 min.	3 (7,1)	39 (92,9)	42 (13,5)		5 (20,0)	20 (80,0)	25 (3,7)	
	Mais de 60 min.	9 (14,1)	55 (85,9)	64 (20,6)		10 (29,4)	24 (70,6)	34 (5,0)	
Carga horária de trabalho semanal									
	20 horas	5 (12,2)	36 (87,8)	41 (13,2)	0,285	14 (63,6)	8 (36,4)	22 (3,3)	0,001
	30 horas	9 (7,6)	109 (92,4)	118 (38,1)		5 (31,3)	11 (68,7)	16 (2,4)	
	40 horas	23 (15,2)	128 (84,8)	151 (48,7)		130 (20,4)	506 (79,6)	636 (94,3)	

a) Total de entrevistados; esse total pode diferir para algumas variáveis, devido a não resposta dos participantes; b) teste qui-quadrado de Pearson; c) teste exato de Fisher.

Entre os agentes penitenciários, os casos positivos para SARS-CoV-2 foram mais frequentes nos indivíduos que apresentaram um sintoma (13,4%), embora 59,7% dos agentes testados com resultado positivo para SARS-Cov-2 não tenham apresentado sintomas. Entre os profissionais de saúde com resultado positivo para o teste, 64,9% não relataram sintomas de COVID-19. Nos agentes penitenciários, observou-se maior proporção de resultados positivos entre os trabalhadores com turnos de 20 horas (63,6%), comparados aos demais regimes de horário de trabalho: 31,3% para 30 horas e 20,4% para 40 horas (p=0,001) (Tabela 2).

**Figura 1:**
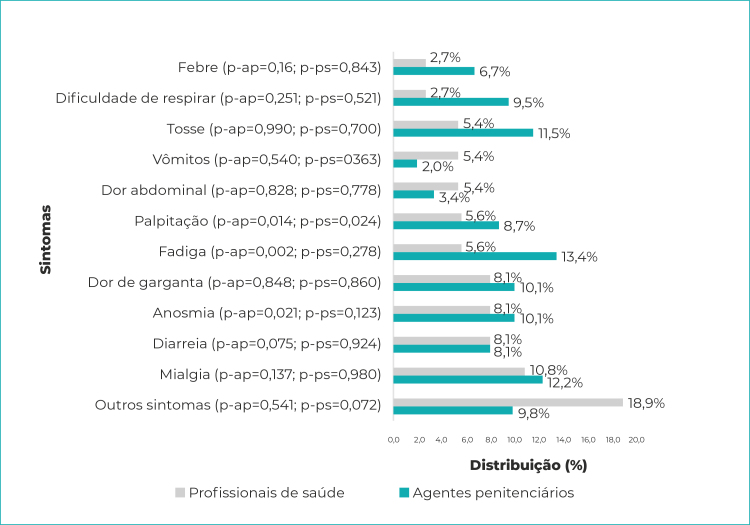
- Distribuição dos sintomas entre profissionais de saúde e agentes penitenciários (n=37 profissionais de saúde e 149 agentes penitenciários) com teste positivo para SARS-CoV-2, Espírito Santo, 2020

Para os trabalhadores que apresentaram resultado do teste positivo, no grupo de profissionais de saúde, o sintoma mais prevalente foi mialgia (10,8%), enquanto para o grupo dos agentes penitenciários foram fadiga (13,4%) e mialgia (12,2%). A positividade do teste mostrou-se associada à presença de palpitação no grupo dos profissionais de saúde (p=0,024), enquanto, nos agentes penitenciários, essa associação esteve associada à presença dos sintomas de anosmia (p=0,021), fadiga (p=0,002) e palpitação (p=0,014) (Figura 1).

Legenda: p-ap = p-valor - agentes penitenciários; p-ps = p-valor - profissionais de saúde.

**Figura 2:**
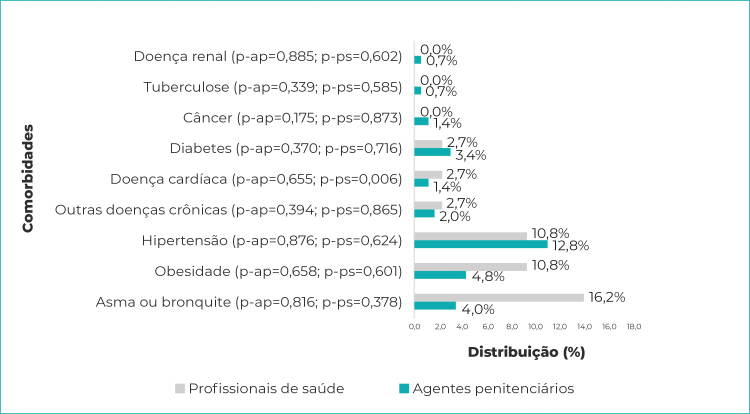
- Distribuição das comorbidades entre profissionais de saúde e agentes penitenciários (n=37 profissionais de saúde e 149 agentes penitenciários) com teste positivo para SARS-CoV-2, Espírito Santo, 2020

A Figura 2 apresenta as comorbidades referidas pelos profissionais de saúde e agentes penitenciários, entre aqueles com resultados positivos para o teste. Nos profissionais de saúde, a distribuição de comorbidades variou de nenhum caso de doença renal, tuberculose e câncer, a 16,2% para asma ou bronquite. Entre os agentes penitenciários, essa distribuição variou de 0,7%, para doença renal e tuberculose, a 12,8% para hipertensão. Finalmente, entre os profissionais de saúde, a presença da doença cardíaca mostrou-se associada à positividade do teste (p=0,006).

Legenda: p-ap = p-valor - agentes penitenciários; p-ps = p-valor - profissionais de saúde.

## DISCUSSÃO

Nos trabalhadores do sistema prisional pesquisados, a prevalência sorológica de infecção por SARS-CoV-2 foi de 11,9% nos profissionais de saúde, e de 22,1% nos agentes penitenciários. Dados do Depen revelam que 24,7% dos testes realizados em servidores prisionais de todo o Brasil apresentaram resultado positivo,^5^ indicando uma pequena diferença em relação aos dados nacionais.

Dados divulgados e coletados pelo CNJ até o final do mês de setembro de 2020 mostram que a taxa de contaminação pelo SARS-CoV-2 entre servidores do sistema prisional do país foi três vezes superior àquela verificada na população geral, haja vista esse grupo contar, registrados, 7.694,5 casos por 100 mil profissionais, enquanto, na população geral, foram contabilizados 2.258,2 casos por 100 mil habitantes. No mesmo período, a taxa de contaminação entre a PPL foi de 3.774,4 casos por 100 mil presos.^9^

 Um estudo realizado com 1.163 trabalhadores de serviços de emergência de Porto Alegre/RS, mostrou uma prevalência de mais de 80% de exposição ao SARS-CoV-2, e uma produção de anticorpos de 5,5% - 1,6 vez superior à proporção de anticorpos produzidos na população do município.^10^

Uma pesquisa em andamento, sobre dados atualizados em 30 de agosto de 2021, com profissionais de saúde que atuam na assistência à COVID-19 nas regiões metropolitanas de Porto Alegre, Fortaleza, Belém, São Paulo e Recife, mostrou que 40,5% desses profissionais apresentaram infecção por COVID-19, e 35,3%, sintomas psiquiátricos.^11^

Para os profissionais de enfermagem, as informações coletadas pelo Conselho Federal de Enfermagem, referentes ao intervalo de tempo entre 20 de março e 28 de maio de 2020, revelaram 17.414 casos suspeitos, 5.732 confirmados e 134 óbitos ocorridos naquele período, no Brasil. Sobre as demais características avaliadas, a região Sudeste apresentou o maior número de casos e de óbitos, a faixa etária com mais casos notificados foi a dos 31 aos 40 anos, e a letalidade maior entre os homens.^12^ São achados que corroboram dados deste estudo, no qual também se observou maior frequência de testes positivos entre os profissionais de saúde da faixa etária de 31 a 40 anos atuantes no sistema prisional. 

Um estudo transversal, realizado em um hospital universitário de São Paulo, avaliou a prevalência da infecção por SARS-Cov-2 entre profissionais de saúde sintomáticos, por meio do teste RT-PCR. Os dados apontaram que 42,4% dos profissionais sintomáticos apresentaram resultado positivo.^13^ Assim como no estudo de São Paulo, a presente pesquisa não encontrou diferença significativa na prevalência de comorbidades entre profissionais com teste positivo ou negativo. Em relação aos sintomas, o mesmo estudo de São Paulo revelou que a anosmia e a dor ocular se apresentaram independentemente associadas à positividade para infecção, enquanto, no presente estudo, anosmia, fadiga e palpitação mostraram-se significantes.

Em março de 2020, a OMS publicou um guia intitulado ‘Preparação, Prevenção e Controle da COVID-19 em prisões e outros locais de detenção’, com o propósito de orientar os países para a tomada de decisões, no que diz respeito ao manejo da COVID-19 nesses ambientes. O documento reforça a importância de cada país se preparar para responder aos possíveis cenários da doença, adaptando-se ao contexto local diante do surgimento dos casos, de maneira a identificar, gerenciar e tratar a doença.^14^

No Brasil, o CNJ publicou sua Recomendação n^o^ 62 de 2020, com medidas preventivas para a propagação da infecção pelo coronavírus nos sistemas de justiça penal e socioeducativo. Ações de desencarceramento e não aprisionamento de indivíduos de grupos de risco (idosos, gestantes e portadores de condições crônicas), ações sanitárias (restrição de visitas, higienização frequente de celas e espaços comuns), triagem das pessoas privadas de liberdade nas entradas das unidades prisionais, bem como de profissionais e visitantes, e isolamento de casos suspeitos ou confirmados, estão entre as principais medidas recomendadas.^15^^,^^16^

Considerando-se ambos os documentos mencionados, a Fundação Instituto Oswaldo Cruz (Fiocruz) elaborou uma cartilha dirigida a gestores e profissionais de saúde do sistema prisional, agregando informações sobre a pandemia da COVID-19 no contexto das instituições de privação de liberdade do Brasil.^17^ Entre as principais contribuições do documento da Fiocruz, destacam-se: (i) a adoção de mudanças de rotina, como suspensão de visitas e separação de presos idosos e portadores de doenças crônicas; (ii) a testagem precoce de qualquer indivíduo com sintoma compatível com a COVID-19; (iii) a notificação de casos suspeitos com sintomas de síndrome gripal no prazo de 24 horas, com incorporação da PPL e trabalhadores do sistema prisional no sistema de vigilância epidemiológica; e (iv) a manutenção dos profissionais de saúde e de segurança atualizados quanto às normativas de acompanhamento e tratamento dos casos da doença.^17^^,^^18^ A cartilha ainda orienta que, para o cuidado amplo em saúde e atenção psicossocial, seja elaborado, pelos níveis locais, um plano de contingenciamento da COVID-19 que inclua desde estratégias de intervenção até a garantia de cuidados especializados no âmbito do Sistema Único de Saúde.^17^

A Secretaria de Estado da Justiça do Espírito Santo elaborou um Plano de Contingência contra a COVID-19, a ser adotado pelas unidades prisionais do estado, com orientações para identificação, prevenção e enfrentamento da COVID-19 no sistema prisional, estabelecendo diretrizes gerais de prevenção e manejo de casos suspeitos e/ou confirmados. O Plano recomenda, entre outras medidas, a triagem de casos suspeitos para a doença mediante aplicação de um questionário e aferição de temperatura de qualquer pessoa que ingresse no sistema prisional, seja PPL, seja trabalhador ou visitante.^19^^,^^20^

É importante salientar que, assim como o ambiente prisional é insalubre e favorece a disseminação do vírus,^4^ as condições de trabalho inadequadas dos profissionais contribuem para agravar esse cenário. Em 2019, o Tribunal de Contas do Estado de São Paulo fiscalizou o sistema prisional paulista, responsável pelo maior quantitativo de presos do país,^21^ e constatou: as unidades prisionais de São Paulo apresentavam uma relação de 9,8 presos para cada agente penitenciário, o dobro do máximo recomendado pelo Conselho Nacional de Política Criminal e Penitenciária, que é o de um agente para cada cinco presos.^22^ Na ocasião, também se verificou que muitas unidades prisionais não contemplavam uma equipe mínima de saúde (18), conforme as recomendações da Portaria Interministerial do Ministério da Saúde e do Ministério da Justiça nº 1.777/2003,^23^ e da Política Nacional de Atenção Integral à Saúde das Pessoas Privadas de Liberdade no Sistema Prisional.

Este estudo apresenta limitações, no que tange ao desenho, não sendo possível determinar a causalidade entre as variáveis estudadas e o resultado positivo do teste, assegurar que os fatores de confusão estejam igualmente distribuídos entre os grupos, ou ainda, retratar a realidade do momento em que a pesquisa foi realizada. Outra limitação deste trabalho se encontra na impossibilidade de distinguir se a infecção por SARS-CoV-2 era ativa ou passada, devido ao resultado único para IgG e IgM apresentado pelo teste. Durante a coleta de dados, nenhum trabalhador foi internado por manifestações graves da COVID-19.

Frente a esse cenário, ressalta-se o desafio vivenciado pelos gestores e profissionais de distintas áreas no enfrentamento da pandemia da COVID-19, evidenciando as fragilidades do sistema penal do país. Todos esses aspectos são, ademais, agravados pela situação do sistema prisional brasileiro, que experimenta condições de precariedade, superlotação, desvalorização e insuficiente quantidade de profissionais, além de outros aspectos. Vale destacar que é dever constitucional do Estado garantir a proteção e tutela dos indivíduos sob sua custódia, bem como fornecer condições de trabalho dignas para seus servidores, com o objetivo de zelar pela manutenção da saúde e bem-estar desses cidadãos.
