# Supplementary material for: Serological prevalence of SARS-CoV-2 infection among prison system workers in the state of Espírito Santo, Brazil, 2020
Source: Epidemiol Serv Saude. 2022 Feb 28;31(1):e2021495. [Article in Portuguese] doi: 10.1590/S1679-49742022000100008 (PMC11342918; doi:10.1590/S1679-49742022000100008)
Supplement: Translation

# Translation: Serological prevalence of SARS-CoV-2 infection among prison system workers in the state of Espírito Santo, Brazil, 2020

**Authors:** Camila Leal Cravo Duque, Laylla Ribeiro Macedo, Ethel Leonor Noia Maciel, Ricardo Tristão-Sá, Erika do Nascimento Bianchi, Adriana Ilha da Silva, Pablo Medeiros Jabor, Cristiana Costa Gomes, Orlei Amaral Cardoso, Pablo Lira, Raphael Lubiana Zanotti, Silvânio José de Souza Magno, Eliana Zandonade

**Affiliations:** 1 Secretaria de Estado da Justiça do Espírito Santo, Diretoria de Saúde do Sistema Penal, Vitória, ES, Brazil; 2 Universidade Federal do Espírito Santo, Laboratório de Epidemiologia, Vitória, ES, Brazil; 3 Universidade Federal do Espírito Santo, Departamento de Medicina Social, Vitória, ES, Brazil; 4 Instituto Jones dos Santos Neves, Coordenação de Geoespacialização, Vitória, ES, Brazil; 5 Secretaria de Estado da Saúde do Espírito Santo, Subsecretaria de Estado de Vigilância em Saúde, Vitória, ES, Brazil; 6 Instituto Jones dos Santos Neves, Diretoria de Integração e Projetos Especiais, Vitória, ES, Brazil; 7 Secretaria de Estado da Justiça do Espírito Santo, Subsecretaria de Planejamento e Controle, Vitória, ES, Brazil; 8 Universidade Federal do Espírito Santo, Departamento de Estatística, Vitória, ES, Brazil

**Keywords:** Prevalence, Coronavirus Infections, Prisons, Occupational Health, Cross-Sectional Studies.

## Abstract

**Objective:**

To estimate the serological prevalence of SARS-CoV-2 infection among prison system workers in the state of Espírito Santo, Brazil, between August-September 2020.

**Methods:**

This was a stratified sample survey, using interviews and serological tests for SARS-CoV-2.

**Results:**

Among the 986 interviewers, the serological prevalence of SARS-CoV-2 infection was 11.9% (95%CI 8.1%;15.7%) in health professionals, and 22.1% (95%CI 18.8%;25.3%) in prison officers. Positivity was more frequent among health professionals in the north of the state (19.7%) and in male prison officers (24.0%). Among seropositive individuals, fatigue was the most frequent symptom in prison agents (13.4%) and myalgia in health professionals (10.8%); and the most prevalent comorbidities among the seropositive individuals were asthma or bronchitis (16.2%), in health professionals, and hypertension in prison officers (12.8%).

**Conclusion:**

The serological prevalence of SARS-Cov-2 infection was higher in prison officers, a finding that can support disease control and prevention actions in this scenario.

## INTRODUCTION

**Table:** 

Study contributions	
Main results	Among 986 prison system workers in Espírito Santo, the serological prevalence of SARS-CoV-2 infection was 11.9% (95%CI 8.1%;15.7%) in health professionals, and 22.1% (95%CI 18.8%;25.3%) in prison officers.
Implications for services	The knowledge of the serological indicators related to workers in the prison environment, enables the managers of these institutions to implement and qualify actions aimed at coping with the COVID -19 pandemic.
Perspectives	Reflections on the weaknesses of the prison system can contribute to the improvement of sanitary conditions observed in prison settings, evidencing the need to ensure the maintenance of the health and well-being of prison staff.

One year after the World Health Organization (WHO) declared the COVID-19 outbreak a pandemic, in March 2020, it could be seen that some population groups, such as black people and individuals with lower income and schooling, have been shown to be more vulnerable to deaths due to SARS-CoV-2.^1^^.^^2^

In addition, individuals in confined spaces with potential agglomeration of people are more exposed to contagion and the spread of the virus.^3^ As such, prison environment provide the ideal conditions that potentially facilitate the spread of COVID-19,^3^ given the precarious situation that Brazilian prison system has been facing, described as poorly maintained places, with little or no ventilation, in addition to the problem of chronic overcrowding.^4^

Data from the Conselho Nacional de Justiça (CNJ), disclosed by the Departmento Penitenciário Nacional (DEPEN), showed that not only persons deprived of liberty (PDL) have been pointed out as the most vulnerable group affected by COVID-19, but also professionals working in these spaces, such as health professionals; and prison and administrative officers.^5^

As of February 22, 2021, 62,459 tests were performed and 15,450 cases of COVID-19 were diagnosed in Brazilian prison system workers, of whom 112 died. Regarding the Federative Units, São Paulo state accounted for the highest number of cases (2,751), while Rio Grande do Sul state had the lowest occurrence.^13^ The South region of Brazil had the lowest percentage of deaths related to the disease, 5.4%, and the Southeast region accounted for 46.6% of deaths from COVID-19 in the country. In the state of Espírito Santo, Southeast region, 835 cases in prison system workers and three deaths due to the disease had been reported until then.^5^

An international study on data from the COVID Prison Project, regarding the period between April 2020 and January 2021, showed that prison staff reported higher rates of COVID-19 compared to the general population, and close to that observed in the PDL from the countries studied.^6^

Thus, it is extremely important to know this reality, in order for the health and/or security institutions to develop strategies aimed at controlling COVID-19 transmission in those places and, consequently, protecting the health of people deprived of liberty, prison staff, health professionals, lawyers or justice professionals, or any individual who enters the prison, in addition to the general community.^7^

This study aimed to estimate the serological prevalence of SARS-CoV-2 infection among the prison system workers in the state of Espírito Santo, Brazil.

## METHODS

This was a cross-sectional study, using data from a population-based survey,^8^ with quota sampling, conducted in prisons in the state of Espírito Santo, stratified according to the categories of workers such as ‘health professionals’ and ‘prison officers’.

The study was conducted between August 31^st^ and September 4^th^, 2020, and had as target population professionals working in the 34 prisons in Espírito Santo state, distributed over 13 municipalities in the state. 441 health professionals and 3,101 prison officers worked in those units.

For the calculation of the sample, we took into consideration the population size described, an expected prevalence of COVID-19 of 10%, sampling error of 2 percentage points and 5% confidence level. The minimum sizes defined for the sample were 293 health professionals and 667 prison officers. They were proportionally calculated according to the number of health professionals and prison officers in each prison unit.

One week before the beginning of this study, according to a list of names provided by the Prison System Administration of the state of Espírito Santo, we selected those who would take part in the study at random, considering the two categories of workers. At this stage of the process, there was around a 40% increase in the number of participants included in the study, in order to compensate for any absences or leave, or alternating work shift.

An application was developed on the ArcGIS Online Platform software, in order to be installed on mobile devices and it could be used offline, which allowed data collection in the prisons. At the end of each application day, the devices were connected to the internet and the technical team immediately received the data package collected on that journey.

A research monitoring panel was developed for each of the prisons, in order to monitor which units were close to or had already achieved the goals for each of the profiles addressed in the survey - prison officers and health professionals working in the state prison system.

The interviewers were trained in the performance of blood test, which was performed with a blood sample collected using digital puncture. Immunochromatographic rapid test for detecting IgM and IgG antibodies, MedLevensohn brand, Agência Nacional de Vigilância Sanitária (Anvisa) registration No. 80560310056, was used. The result was estimated based on the observation of the markers, according to the amount of time specified by the manufacturer. In case of doubts regarding the interpretation, the tests were repeated and forwarded to the medical team responsible for the research. The test was considered positive - IgG or IgM; however, information on which of the two markers had a positive result was not available to the authors.

The interviewers received all personal protective equipment (PPE) recommended by the Anvisa for possible contact with people with COVID-19, in addition to other necessary items, such as cell phones and alcohol, and also other materials used in serological surveys, provided by the Espírito Santo State Department of Health.

In addition to COVID-19 testing, information was collected from the participants using a face-to-face interview with closed-ended questions. As such, the following variables were selected and categorized:

- sex (male; female);
- age group (in years: up to 30; 31 to 40; 41 to 50; 51 and more);
- schooling (complete high school; incomplete higher education; complete higher education; master’s degree);
- race/skin color (Asian; White; Indigenous; Brown; Black);
- number of COVID-19 symptoms in the last 15 days (none; 1; 2; 3 or more);
- access to the health services in the last 15 days (no; yes);
- hand hygiene habits (no; yes);
- use of public transportation to commute to work (no; yes);
- frequency of daily use of public transportation to commute to work (does not use; up to 3 times a day; 4 times a day or more);
- average daily commute time using public transportation (does not use; less than 30 minutes; between 30 and 60 minutes; more than 60 minutes); and
- workload (in hours: 20; 30; 40).

The information was stored in a database, and the statistical analysis was performed using the Statistical Package for the Social Science (SPSS), version 20.0. Prevalence and respective 95% confidence intervals (95%CI) were estimated. The differences between exposure variables and COVID-19 seropositivity, stratified for health professionals and prison officers, was analyzed using Pearson’s chi-square test, or Fisher’s exact test. The level of significance adopted was 5%.

All individuals selected for the sample in the prison units were informed about the objective of this study, risks and benefits involved, along with advice on preventive measures. The material for serology tests and data were collected after the participants signed the Free and Informed Consent Form. All individuals who had been tested, received the result a few minutes after its performance. Positive cases were reported and referred to the Municipal Health Services for clinical evaluation. All biosafety measures were taken in order to ensure the health of field workers. The study was approved by the Human Research Ethics Committee, Center for Health Sciences of the Universidade Federal do Espírito Santo (CEP/CCS/UFES): Opinion No. 4,209,127, issued on August 12, 2020.

## RESULTS

986 individuals working in the prison system in the state of Espírito Santo took part in the survey: 311 health professionals and 675 prison officers. There were no refusals to participate in the study.

The majority of the health professionals who took part in the study worked in prisons in the metropolitan region of Vitória, capital city of Espírito Santos (61.7%), closed regime (90.7%), were female (76.8%), aged between 31 and 40 years (41.3%), had complete higher education (43.5%) and self-reported Brown race/skin color (44.8%). Among prison officers, most of them worked in prisons in the metropolitan region of Vitória (59.4%), closed regime (89.5%), were male (76.1%), aged between 31 and 40 years (54.8%), had complete higher education (45.5%) and self-reported Brown race/skin color (49.9%) (Table 1).

**Table 1:** - Distribution of sociodemographic characteristics of health professionals and prison officers, according to the result of SARS-CoV-2 rapid test, Espírito Santo state, 2020

Variable	Category	Health professional N=311^a^				Prison officer N=675^a^			
		Positive	Negative	Total	p-value^b^	Positive	Negative	Total	p-value^b^
		n (%)	n (%)	n (%)		n (%)	n (%)	n (%)	
Prison location (region)									
	Metropolitan	14 (7.3)	178 (92.7)	192 (61.7)	0.006	86 (21.4)	315 (78.6)	401 (59.4)	0.178
	North	18 (19.7)	73 (80.3)	91 (29.3)		50 (25.9)	143 (74.1)	193 (28.6)	
	South	5 (17.9)	23 (82.1)	28 (9.0)		13 (16.0)	68 (84.0)	81 (12.0)	
Prison regime									
	Closed	35 (12.4)	247 (87.6)	282 (90.7)	0.382^c^	139 (23.0)	465 (77.0)	604 (89.5)	0.086
	Open	2 (6.9)	27 (93.1)	29 (9.3)		10 (14.1)	61 (85.9)	71 (10.5)	
Sex									
	Female	31 (13.0)	208 (87.0)	239 (76.8)	0.287	24 (15.0)	136 (85.0)	160 (23.9)	0.022
	Male	6 (8.3)	66 (91.7)	72 (23.2)		123 (24.0)	388 (76.0)	511 (76.1)	
Age group (in years)									
	≤30	13 (11.3)	102 (88.7)	115 (37.1)	0.543^c^	9 (19.1)	38 (80.9)	47 (7.0)	0.665
	31-40	14 (10.9)	114 (89.1)	128 (41.3)		77 (20.9)	292 (79.1)	369 (54.8)	
	41-50	9 (17.7)	42 (82.3)	51 (16.4)		50 (24.0)	158 (76.0)	208 (30.9)	
	≥51	1 (6.3)	15 (93.7)	16 (5.2)		13 (26.5)	36 (73.5)	49 (7.3)	
Schooling									
	Complete high school	11 (20.0)	44 (80.0)	55 (17.8)	0.012^c^	52 (28.7)	129 (71.3)	181 (26.9)	0.025
	Incomplete higher education	3 (15.8)	16 (84.2)	19 (6.1)		15 (16.3)	77 (83.7)	92 (13.6)	
	Complete higher education	14 (10.4)	121 (89.6)	135 (43.5)		64 (20.9)	243 (79.1)	307 (45.5)	
	Master’s degree	8 (7.9)	93 (92.1)	101 (32.6)		17 (18.1)	77 (81.9)	94 (14.0)	
Race/skin color									
	Yellow	1 (33.3)	2 (66.7)	3 (1.0)	0.022^c^	(0.0)	4 (100.0)	4 (0.6)	0.472^c^
	White	7 (5.5)	120 (94.5)	127 (41.0)		45 (20.1)	179 (79.9)	224 (33.2)	
	Indigenous	(0.0)	(0.0)	(0.0)		(0.0)	4 (100.0)	4 (0.6)	
	Brown	25 (18.0)	114 (82.0)	139 (44.8)		81 (24.0)	256 (76.0)	337 (49.9)	
	Black	4 (9,7)	37 (90,3)	41 (13,2)		23 (21,7)	83 (78,3)	106 (15,7)	

a) Total number of respondents; this total may be different for some variables, due to non-response; b) Pearson chi-square test; c) Fisher’s exact test.

The prevalence of positive cases was 11.9% (95%CI 8.1%;15.7%) among health professionals, and 22.1% (95%CI 18.8%;25.3%) among prison officers.

Positive cases for SARS-CoV-2 were more frequent among health professionals in the north region of Espírito Santo (19.7%) and in male prison officers (24.0%). In both groups of workers, the outcome was associated with lower education (complete high school) (Table 1).

Most health professionals and prison officers had no symptoms (67.5% of health professionals and 64.9% of prison officers) and had not sought health care in the last 15 days (77.5% of health professionals and 83.8% of prison officers) did not have comorbidities (73.6% of health professionals and 77.8% of prison officers), reported hand hygiene habits (99.0% of health professionals and 98.2% of prison agents), did not use public transportation to commute to work (61.0% of health professionals and 90.1% of prison officers) and had a weekly workload of 40 hours (48.7% of health professionals and 94.3% of prison officers) (Table 2).

**Table 2:** - Distribution of the number of symptoms, comorbidities, health service seeking and handwashing habits among health professionals and prison officers, according to the result of SARS-CoV-2 rapid test, Espírito Santo state, 2020

Variable	Category	Health professional N=311^a^				Prison officer N=675^a^			
		Positive	Negative	Total	p-value^b^	Positive	Negative	Total	p-value^b^
		n (%)	n (%)	n (%)		n (%)	n (%)	n (%)	
Number of symptoms in the last 15 days									
	None	24 (11.4)	186 (88.6)	210 (67.5)	0.342^c^	89 (20.3)	349 (79.7)	438 (64.9)	0.026
	1	7 (17.9)	32 (82.1)	39 (12.5)		20 (17.4)	95 (82.6)	115 (17.0)	
	2	1 (3.6)	27 (96.4)	28 (9.0)		15 (32.6)	31 (67.4)	46 (6.8)	
	3	1 (7.7)	12 (92.3)	13 (4.2)		10 (28.6)	25 (71.4)	35 (5.2)	
	≥4	4 (19.1)	17 (80.9)	21 (6.8)		15 (36.6)	26 (63.4)	41 (6.1)	
Access to health services in the last 15 days									
	No	30 (12.6)	208 (87.4)	238 (77.5)	0.580	126 (22.3)	439 (77.7)	565 (83.8)	0.782
	Yes	7 (10.1)	62 (89.9)	69 (22.5)		23 (21.1)	86 (78.9)	109 (16.2)	
Number of comorbidities									
	None	23 (10.1)	206 (89.9)	229 (73.6)	0.150^c^	117 (22.3)	408 (77.7)	525 (77.8)	0.972^c^
	1	12 (18.8)	52 (81.2)	64 (20.6)		25 (21.4)	92 (78.6)	117 (17.3)	
	2	1 (6.7)	14 (93.3)	15 (4.8)		5 (21.7)	18 (78.3)	23 (3.4)	
	3	1 (33.3)	2 (67.7)	3 (1.0)		1 (14.3)	6 (85.7)	7 (1.0)	
	≥4	- (0.0)	- (0.0)	- (0.0)		1 (33.3)	2 (66.7)	3 (0.5)	
Hand hygiene habits									
	No	- (0.0)	3 (100.0)	3 (1.0)	0.521^c^	1 (8.3)	11 (91.7)	12 (1.8)	0.247^c^
	Yes	37 (12.1)	269 (87.9)	306 (99.0)		148 (22.3)	515 (77.7)	663 (98.2)	
Use of public transportation to commute to work									
	No	24 (12.7)	165 (87.3)	189 (61.0)	0.817	130 (21.4)	477 (78.6)	607 (90.1)	0.101
	Yes	13 (10.7)	108 (89.3)	121 (39.0)		18 (26.9)	49 (73.1)	67 (9.9)	
Frequency of daily use of public transportation to commute to work									
	Does not use	24 (12.6)	166 (87.4)	190 (61.1)	0.812^c^	131 (21.6)	477 (78.4)	608 (90.1)	0.178
	4 times or more	10 (11.5)	77 (88.5)	87 (28.0)		9 (20.9)	34 (79.1)	43 (6.4)	
	Up to 3 times	3 (8.8)	31 (91.2)	34 (10.9)		9 (37.5)	15 (62.5)	24 (3.5)	
Average daily commute time using public transportation									
	Does not use	24 (12.6)	166 (87.4)	190 (61.1)	0.641^c^	131 (21.6)	477 (78.4)	608 (90.1)	0.506^c^
	Less than 30 minutes	1 (6.7)	14 (93.3)	15 (4.8)		3 (37.5)	5 (62.5)	8 (1.2)	
	Between 30 and 60 minutes	3 (7.1)	39 (92.9)	42 (13.5)		5 (20.0)	20 (80.0)	25 (3.7)	
	More than 60 minutes	9 (14.1)	55 (85.9)	64 (20.6)		10 (29.4)	24 (70.6)	34 (5.0)	
Weekly workload									
	20 hours	5 (12.2)	36 (87.8)	41 (13.2)	0.285	14 (63.6)	8 (36.4)	22 (3.3)	0.001
	30 hours	9 (7.6)	109 (92.4)	118 (38.1)		5 (31.3)	11 (68.7)	16 (2.4)	
	40 hours	23 (15.2)	128 (84.8)	151 (48.7)		130 (20.4)	506 (79.6)	636 (94.3)	

a) Total number of respondents; this total may be different for some variables, due to non-response; b) Pearson chi-square test; c) Fisher’s exact test.

Among prison officers, positive cases for SARS-CoV-2 were more frequent in individuals who presented one symptom (13.4%), although 59.7% of the prison officers who tested positive for SARS-Cov-2 did not present any symptoms. Among health professionals who had positive test results, 64.9% did not report covid-19 symptoms. Regarding prison officers, a higher proportion of positive results was observed among individuals working 20-hour shifts (63.6%), compared to other working time regimes: 31.3% for 30 hours and 20.4% for 40 hours (p=0.001) (Table 2).

With regard to workers who had positive test results in the group of health professionals, the most prevalent symptom was myalgia (10.8%), while in the group of prison officers, fatigue (13.4%) and myalgia (12.2%) were the most frequent symptoms. Test positivity was associated with the presence of heart palpitations in the group of health professionals (p=0.024), while among prison officers, it was associated with the symptoms of anosmia (p=0.021), fatigue (p=0.002) and palpitations (p=0.014) (Figure 1).

**Figure 1:** - Distribution of symptoms among health professionals and prison officers (n=37 health professionals and 149 prison officers) who tested positive for SARS-CoV-2, Espírito Santo state, 2020

Legend: p-po = p-value - prison officers; p-hp = p-value- health professionals.

Figure 2 shows the comorbidities reported by health professionals and prison officers, among those who had positive test results. Regarding health professionals, the distribution of comorbidities ranged from no cases of kidney disease, tuberculosis and cancer to 16.2% of asthma or bronchitis. Among prison officers, this distribution ranged from 0.7% of kidney disease and tuberculosis to 12.8% of hypertension. Finally, among health professionals, the presence of heart disease was associated with test positivity (p=0.006).

**Figure 2:** - Distribution of comorbidities among health professionals and prison officers (n=37 health professionals and 149 prison officers) who tested positive for SARS-CoV-2, Espírito Santo state, 2020

Legend: p-po = p-value - prison officers; p-hp = p-value- health professionals.

## DISCUSSION

Among the prison staff who took part in this study, the sorological prevalence of SARS-CoV-2 infection was 11.9% in health professionals, and 22.1% in prison officers. Data from DEPEN revealed that 24.7% of the tests performed on prison staff nationwide showed a positive result,^5^ indicating a small difference regarding national data.

Data disclosed and collected by the CNJ (Conselho Nacional de Justiça) until the end of September 2020 showed that the contamination rate of SARS-CoV-2 among prison staff in the country was three times as high as that found in the general population, given that this group recorded 7,694.5 cases per 100,000 professionals, while in the general population, there were 2,258.2 cases per 100,000 inhabitants. In the same period, the contamination rate among the PDL was 3,774.4 cases per 100,000 prisoners.^9^

A study conducted with 1,163 emergency service workers in Porto Alegre, capital city of Rio Grande do Sul, showed a prevalence of more than 80% exposure to SARS-CoV-2, and an antibody production of 5.5% - 1.6 times as high as the proportion of antibodies developed by the municipal population.^10^

An ongoing research on data updated on August 30^th^, 2021, conducted with health professionals providing care to those with COVID-19, in the metropolitan regions of Porto Alegre, Fortaleza, capital city of Ceará, Belém, capital city of Pará, São Paulo, and Recife, capital city of Pernambuco, showed that 40.5% of these professionals had COVID-19 infection and 35.3% psychiatric symptoms.^11^

For nursing professionals, the information collected by the Federal Nursing Council, regarding the time interval between March 20^th^ and May 28^th^, 2020, showed 17,414 suspected and 5,732 confirmed cases, and 134 deaths occurred in that period in Brazil. With regard to other characteristics evaluated, the Southeast region presented the highest number of cases and deaths, the age group with the most reported cases was 31-40, and the highest case fatality ratio among men.^12^ These findings corroborate data from this study, in which a higher frequency of positive test results among health professionals aged 31 to 40 years working in the prison system was also observed.

A cross-sectional study conducted at a university hospital in São Paulo assessed the prevalence of SARS-Cov-2 infection among symptomatic health professionals using the RT-PCR test. Data showed that 42.4% of the symptomatic professionals presented a positive result.^13^ As in the study conducted in São Paulo, the present study did not find a significant difference in the prevalence of comorbidities among professionals with positive or negative test results. Regarding symptoms, the same study conducted in São Paulo showed that anosmia and eye pain were independently associated with infection positivity, while in this study, anosmia, fatigue and palpitation were significant.

In March 2020, WHO published a guidance entitled ‘Preparedness, Prevention and Control of COVID-19 in prisons and other places of detention’, aimed to assist countries in developing specific plans for these detention settings to deal with COVID-19. The document reinforces the importance for each country to be prepared to respond to possible scenarios for the disease. Therefore, countries should adjust and tailor their approach to the local context, in order to identify, manage and treat the disease.^14^

In Brazil, the CNJ published its Recommendation No. 62, 2020, with preventive measures against the spread of coronavirus infection in criminal and socio-educational justice systems. Actions aimed at release and non-imprisonment of individuals in risk groups (the elderly, pregnant women and people with chronic conditions), health actions (restriction of visits, frequent hygiene of cells and common spaces), screening persons deprived of liberty at prison entrance, as well as professionals and visitors, and isolation for suspected or confirmed COVID-19 cases, are among the main recommended measures.^15^^.^^16^

Taking into consideration both aforementioned documents, the Fundação Instituto Oswaldo Cruz (Fiocruz) has developed a booklet addressed to health professionals and managers working in the prison system, adding information about the COVID-19 pandemic in the context of Brazilian prison system.^17^ Among the main contributions of Fiocruz’s document, the following measures stand out: (i) the adoption of changes in daily routine, such as suspension of visits and separation of elderly prisoners and people with chronic diseases; (ii) early COVID-19 testing in individuals with compatible symptoms; (iii) the notification of suspected COVID-19 cases with flu-like symptoms within 24 hours, and the inclusion of PDL and prison staff in the epidemiological surveillance system; and, finally, (iv) keeping health professionals and prison officers informed about the regulations for the monitoring and treatment of cases of the disease.^17^^.^^18^ The booklet also advises that for comprehensive health care and psychosocial support, a COVID-19 contingency plan should be developed at local levels, including both intervention strategies and the guarantee of specialized health care within the scope of the Brazilian National Health System.^17^

The State Secretariat of Justice of Espírito Santos has developed a COVID-19 Contingency Plan to be adopted by the prison units in the state, with recommendations for identifying, preventing and coping with COVID-19 in the prison system, establishing general guidelines for the prevention and management of suspected and/or confirmed cases. The Plan recommends, among other measures, the screening of suspected COVID-19 cases using a questionnaire and temperature measurement of people at prison entrance such as PDL, prison staff and visitors.^19^^.^^20^

It is worth highlighting that, the prison environment is unhealthy and favors the spread of the virus,^4^ as well as the inadequate working conditions of prison staff contribute to aggravate this scenario. In 2019, the Tribunal de Contas do Estado de São Paulo inspected the São Paulo prison system, responsible for the largest number of prisoners in the country,^21^ and found: the inmate-to-prison officer ratio was 9.8 to 1, in prisons in São Paulo, twice as high as the maximum recommended by the Conselho Nacional de Política Criminal e Penitenciária, which is an inmate-to-prison officer ratio of 5 to 1.^22^ In the same period, it could be seen that many prisons did not have a team with a minimum number of health professionals (18), according to the recommendations of the Interministerial Ordinance of the Ministry of Health and Ministry of Justice, No. 1,777/2003,^23^ and the National Policy for Comprehensive Health Care of People Deprived of Liberty in the Prison System.

This study presents limitations regarding the design, and it is not possible to determine the causality between the variables studied and the positive test result to ensure that the confounding factors are equally distributed among the groups, or even to portray the reality of the moment when the research was conducted. Another limitation of this study lies in the impossibility of distinguishing whether SARS-CoV-2 was active or past infection, due to a single test result for IgG and IgM antibodies. During data collection, no worker was hospitalized due to severe manifestations of COVID-19.

Given this scenario, it is worth highlighting the challenge experienced by managers and professionals from different areas in coping with the COVID-19 pandemic, showing the weaknesses of the country’s penal system. All these aspects have been aggravated by the precarious situation that Brazilian prison system has been facing, in addition to overcrowding, devaluation and insufficient number of professionals, among other aspects. It is worth mentioning that the Brazilian state has a constitutional duty to guarantee the protection of individuals in its custody, as well as to provide adequate working conditions for its employees, with the objective of ensuring the maintenance of the health and well-being of these citizens.
